# Acute contrecoup epidural hematoma that developed without skull fracture in two adults: two case reports

**DOI:** 10.1186/s13256-018-1676-1

**Published:** 2018-06-14

**Authors:** Shunpei Andoh, Chie Matsuura, Yuuki Sakaeyama, Shinichi Okonogi, Yasuhiro Node, Hiroyuki Masuda, Kousuke Kondo, Naoyuki Harada, Masaaki Nemoto, Nobuo Sugo

**Affiliations:** Department of Neurosurgery (Omori), School of Medicine, Faculty of Medicine, Toho University, 6-11-1, Omori-nishi, Ota-ku, Tokyo, 143-8541 Japan

**Keywords:** Acute epidural hematoma, Bone fracture, Contrecoup injury

## Abstract

**Background:**

The incidence of acute epidural hematoma not accompanied by fracture is low, and it mostly occurs right below the impact point in children. Acute epidural hematoma on the contralateral side of the impact point without fracture is very rare.

**Case presentation:**

Case 1: a 52-year-old Japanese woman fell and was bruised in the left occipital region, and acute epidural hematoma developed in the right frontal region. No fracture line was observed in the right frontal region on head computed tomography or during surgery, and the source of bleeding was the middle meningeal artery. Case 2: a 56-year-old Japanese man fell down the stairs and was bruised in the right occipital region, and acute epidural hematoma developed in the right occipital supra- and infratentorial regions and left frontal region. Separation of the lambdoid suture was noted in the right occipital region, but no fracture line was present in the left frontal region on either head computed tomography or during surgery, and the source of bleeding was the middle meningeal artery.

**Conclusions:**

Two rare cases of frontal contrecoup acute epidural hematoma without facture near the hematoma were reported. It is possible that the dura mater detaches from the inner surface of the skull due to cavitation theory-related negative pressure and blood vessels in the dura mater are damaged, causing contrecoup acute epidural hematoma even though no fracture occurs, for which careful course observation is necessary.

## Background

The incidence of traumatic acute epidural hematoma (AEDH) is high in young people, and hematoma is formed right below the impact point accompanied by skull fracture in most cases [1–4]. In contrast, the incidence of AEDH not accompanied by fracture is low and most cases occur right below the impact point in children [2, 3]. We encountered two adults in whom rare contrecoup AEDH not accompanied by skull fracture developed. We report the cases with a literature review.

## Case presentation

### Case 1: a 52-year-old Japanese woman

**Fig. 1:**
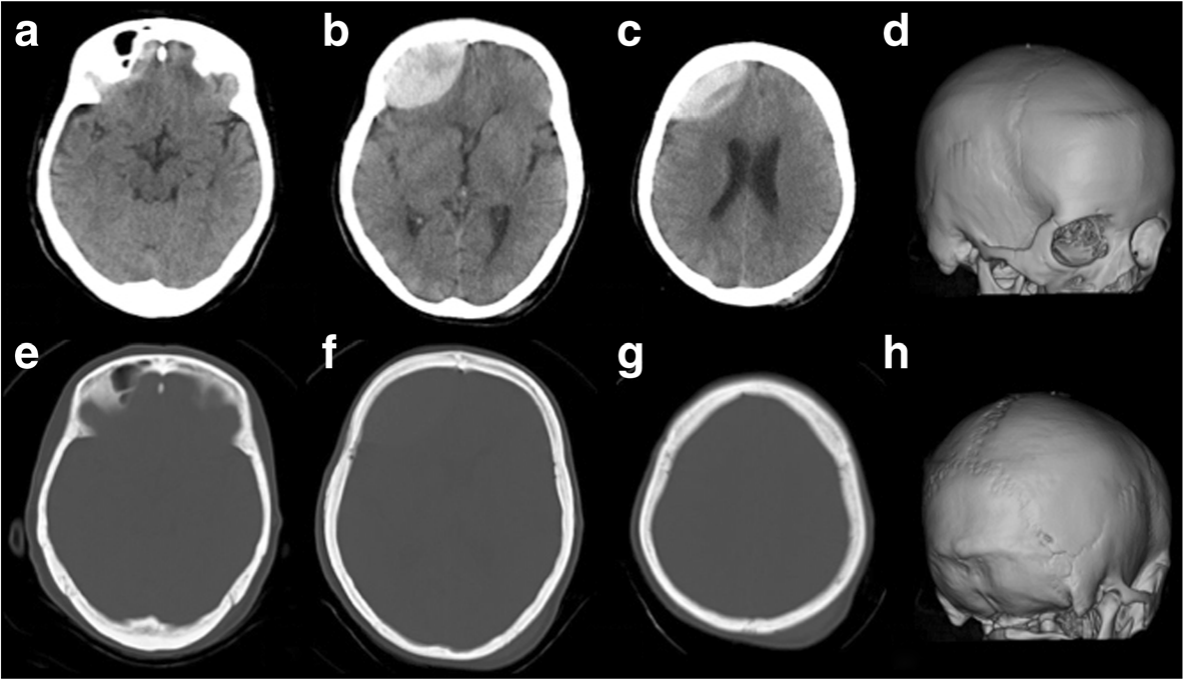
Head computed tomography (case 1). Computed tomography at 150 min after the injury showed acute epidural hematoma in the right frontal region (**a**-**c**) and no evidence of bone fracture (**d**-**h**)

**Fig. 2:**
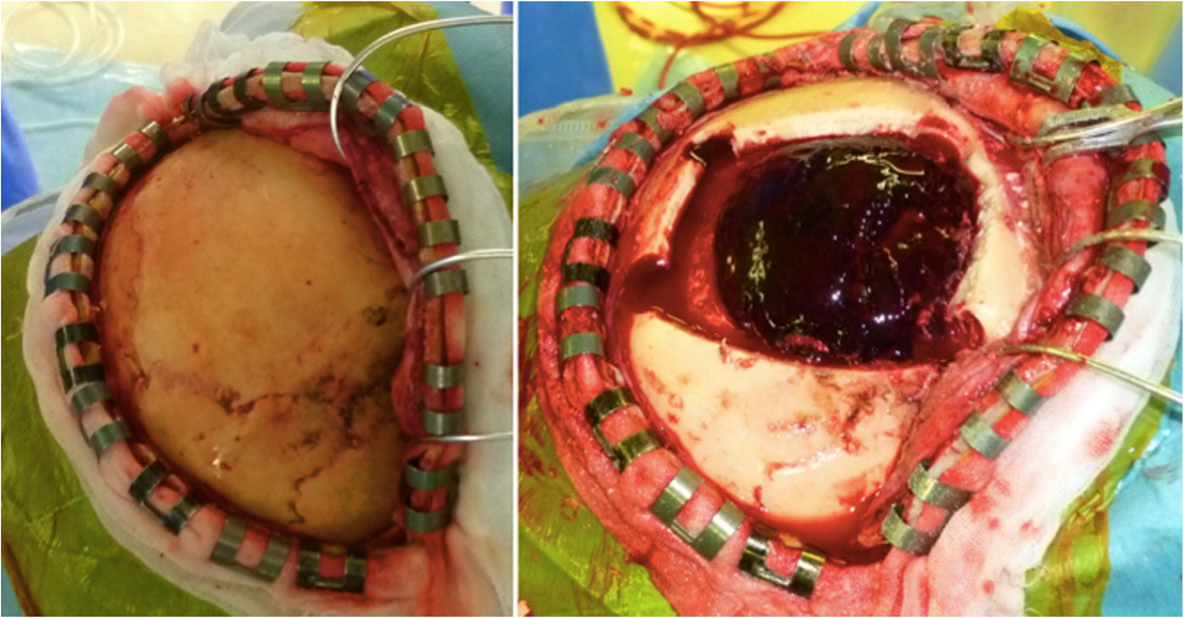
Acute epidural hematoma without bone fracture (case 1). The site of bleeding was traced to the right middle meningeal artery

Our patient fell when getting out of a car and was bruised in the left occipital region. She visited a physician, and her Glasgow Coma Scale (GCS) was 15 with no other neurological abnormality. She was diagnosed with AEDH in the right frontal region on head computed tomography (CT) 2.5 hours after injury (Fig. 1) and transferred to our hospital. On visual examination of her head, contusion was present in the left occipital region, but no traumatic change was noted in the right frontal region. Since disturbance of consciousness rapidly aggravated to GCS10 (E3V3M4), emergency craniotomy was performed to remove hematoma. No fracture line was observed in the right frontal region on preoperative CT or during surgery, and the source of bleeding was the middle meningeal artery (Fig. 2). The postoperative course was favorable, and our patient was discharged without any neurological abnormality 15 days after surgery.

### Case 2: a 56-year-old Japanese man

**Fig. 3:**
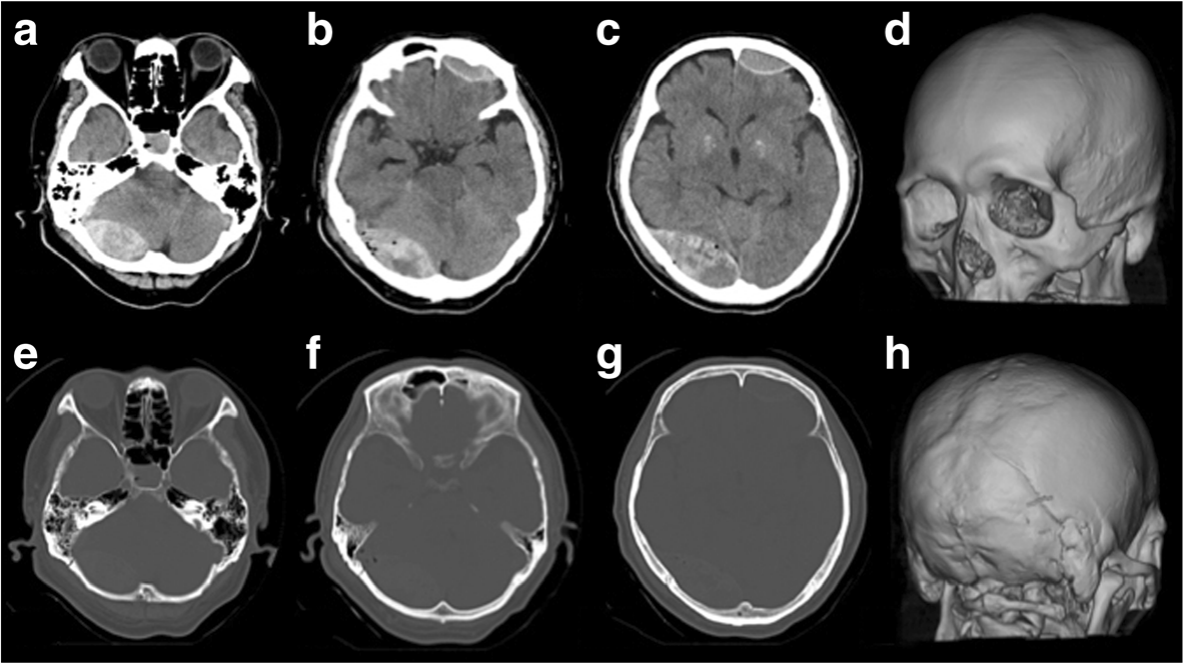
Head computed tomography (case 2) on admission. Computed tomography showed acute epidural hematoma in the right occipital, right suboccipital, and left frontal region (**a**-**c**). There was a separated skull fracture on the lambdoid suture (**d**-**h**)

**Fig. 4:**
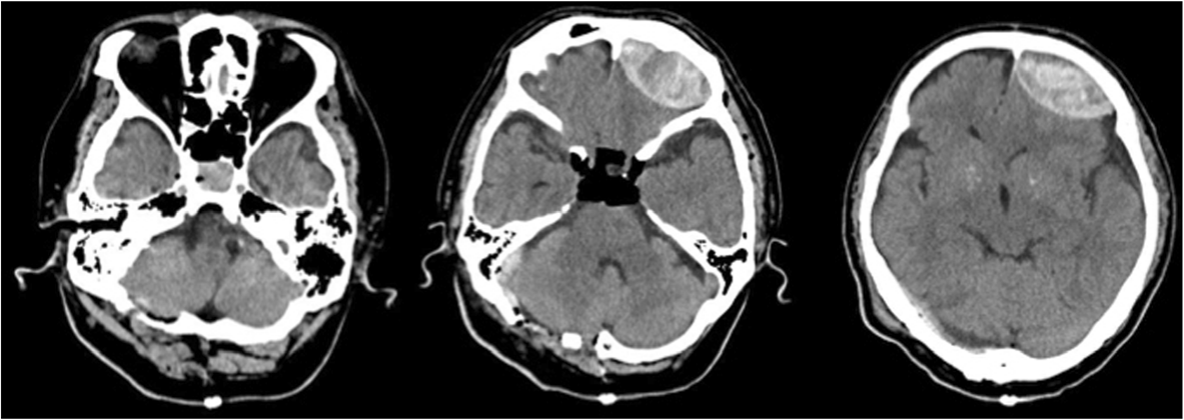
Head computed tomography after surgery. Computed tomography showed enlargement of the left frontal acute epidural hematoma

**Fig. 5:**
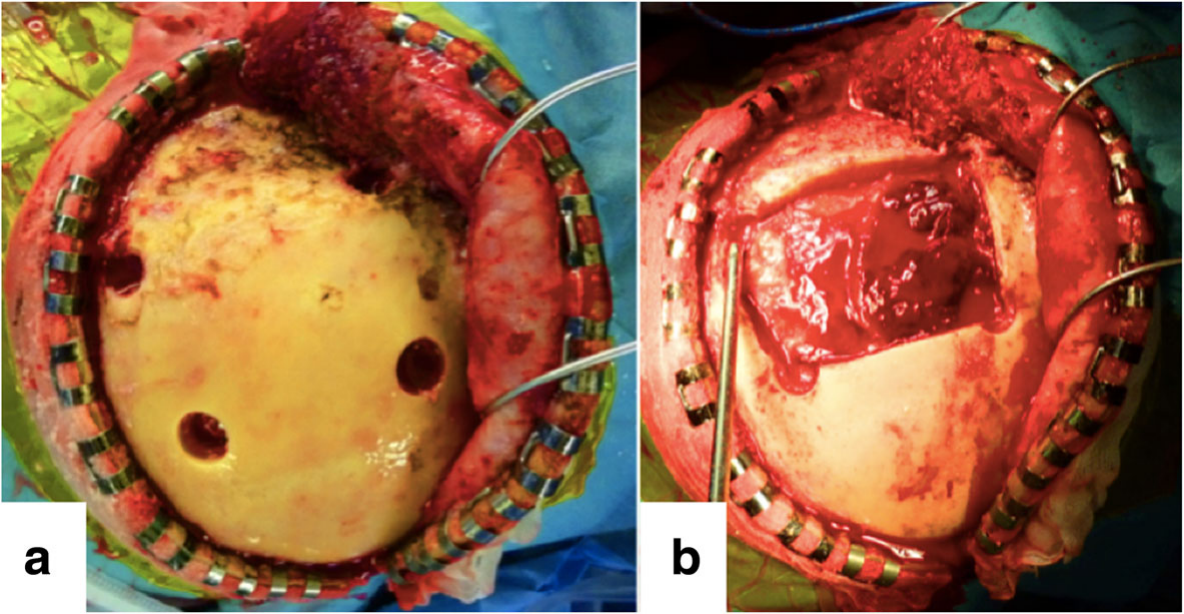
Intraoperative photograph. **a** Acute epidural hematoma without bone fracture (case 2). **b** The site of bleeding was traced to the left middle meningeal artery

The patient fell down the stairs after drinking alcohol and was bruised in the right occipital region. On the first examination by a physician, his GCS was 15 and AEDH extending over the supra- and infratentorial regions in the right occipital region was noted, and the patient was transferred to our hospital. At the time of arrival, his GCS was 6 (E1V1M4) and the AEDH enlarged on CT. Moreover, a new AEDH appeared in the left frontal region (Fig. 3). On visual examination, abrasion and subcutaneous hemorrhage were noted in the right occipital region, but there was no abnormal traumatic finding in the left frontal region. Emergency craniotomy was performed to remove the AEDH in the right occipital region. Separation of the right lambdoid suture was noted on preoperative CT and during surgery, and the source of bleeding was the transverse sinus. Since AEDH in the left frontal region was enlarged on CT immediately after surgery, craniotomy was subsequently performed to remove this hematoma (Fig. 4). The frontal bone was not fractured on preoperative CT or during surgery (Fig. 5a). The source of bleeding was the middle meningeal artery (Fig. 5b). The postoperative course was favorable, and our patient was discharged without any neurological abnormality 28 days after surgery.

## Discussion

AEDH accounts for 1–3% of all head injury cases [3], and is a common disease in neurosurgery and emergency medicine. It develops right below the impact point accompanied by linear fracture in most cases [1–4], and reportedly, cases not accompanied by fracture account for 10–20% [2, 3]. Generally, the incidence of AEDH is high in people in their 10–20s and low in infants aged 2 years or younger and the elderly. On the other hand, the incidence of AEDH without fracture tends to be higher in children [2, 3]. Fibrous tissue is replaced by bone tissue in the bone suture region by about 22 years old, and the inner table of the skull is readily distorted by traumatic impact causing detachment of the dura mater because the skull is elastic. Subsequently, blood vessels feeding the dura mater and small blood vessels and venous sinus present between the dura mater and skull are readily damaged right below the impact point, which may cause epidural hematoma formation even though there is no accompanying fracture [3].

To the best of our knowledge, 21 cases of contrecoup AEDH without fracture have been reported, including our patients (Table 1). The age was in their 50s in ten cases, being the most frequent, and there were only a few patients younger than 20 years old and older than 60 years old. The temporal region was injured in many normal AEDH cases, whereas the occipital (ten cases) and frontal (five cases) regions were injured in the contrecoup AEDH cases, accounting for more than 70%.

**Table 1:** Summary of acute epidural hematoma caused by contrecoup injury without bone fracture

Case	Author (year)	Age	Sex	impact point	fracture of impact side	coup injury	operation for coup injury	site of contrecoup EDH	operation for contrecoup EDH	injury vessels	Outcome
1	Ikeda et al. (1980) [8]	5	F	Rt T	―	―	―	Lt SO, bil O	+	confluens sinuum	GR
2	Takada et al. (2010) [9]	9	M	Rt O	―	―	―	Lt F	―	unknown	GR
3	Okita et al. (1988) [10]	18	F	Rt O	+	EDH	―	Lt F	+	unknown	GR
4	Hirai et al. (2004) [11]	20	F	Rt T	+	EDH	+	Lt T	+	nc	GR
5	Balasubramaniam and Ramesh (1991) [12]	21	M	Rt P	+	EDH	+	Lt F	+	small dural vessels	GR
6	Ikeda et al. (1980) [8]	28	M	Rt F	nc	―	―	Lt SO, Lt O	+	nc	GR
7	Bucci et al. (1986) [13]	34	F	Lt F	―	―	―	Lt O	+	nc	dead
8	Abe et al. (1988) [7]	36	M	Rt F	+	―	―	bil SO, Lt O	+	Lt TS	dead
9	Yanagawa et al. (1998) [6]	39	F	Rt facial	+	―	―	Lt SO, Lt O	+	Lt TS	GR
10	Shigemori et al. (1985) [5]	43	M	Rt FT	+	―	―	Rt SO, Rt O	+	Rt TS	GR
11	Mishra and Mohanty (2001) [14]	50	M	Lt FP	+	contusion	―	Rt FP	+	nc	GR
12	Mitsuyama et al. (2004) [1]	50	F	Lt P	+	EDH	+	Rt F	―	unknown	GR
13	Okamoto et al. (1983) [15]	51	F	O	―	―	―	Lt F	+	unknown	GR
14	Miyazaki et al. (1995) [2]	52	F	Lt O	+	EDH	―	Rt F	+	small dural vessels	GR
15	Okinaga et al. (2002) [16]	55	F	Rt O	+	EDH	+	bil F	+	SSS	GR
16	Nakagawa et al. (1990) [17]	57	F	Lt T	+	EDH	+	Rt T	+	unknown	GR
17	Hamasaki et al. (1987) [18]	58	F	Rt O	+	contusion	+	Rt F	―	unknown	dead
18	Motohashi et al. (2000) [3]	59	F	O	+	―	―	Lt F	―	unknown	GR
19	Sato et al. (2009) [19]	68	F	Rt O	+	EDH	―	Lt F	―	unknown	GR
20	Our case	52	F	Lt O	―	―	―	Rt F	+	MMA	GR
21		56	M	Rt O	+	EDH	+	Lt F	+	MMA	GR

*bil* bilateral, *F* frontal, *FT* frontotemporal, *FP* frontoparietal, *GR* good recovery, MMA middle meningeal artery, *nc* no contribution, *O* occipital, *SO* suboccipital, *SSS* superficial saggital sinus, *T* temporal, *TS* transverse sinus

Contrecoup AEDH without fracture occurs through the following two developmental mechanisms: First, AEDH formed in the occipital region is considered due to skull development. The occipital bone develops from two types of tissue, membranous and cartilaginous tissues, and the transverse sinus is present in the boundary between these tissues. Thus, this region is readily deformed or distorted, and reported to be a region with reduced resistance against external forces [5]. In previous reports with detailed descriptions, the injured blood vessel of AEDH in the occipital region caused by contusion of the frontal region was the transverse sinus in all cases, supporting the mechanism described above. Second, the developmental mechanism of AEDH formed in the frontal region is explained with the cavitation theory proposed by Word *et al.* When acceleration is added to the head, the hard and light skull readily moves and stops, whereas soft and heavy brain tissue does not readily move or stop. The skull and brain tissue moves differently, generating a spatial gap between the two tissues. When the head gets a bruise, the skull rapidly stops but movement of brain tissue continues due to inertia, separating the brain tissue from the skull. Subsequently, negative pressure is generated between the two tissues and causes detachment of the dura mater. Regarding the frontal region, it has been reported that anatomically, the dura mater is readily detached [6], and a 1.6 times higher negative pressure is loaded compared with that in the occipital region because inflow of cerebrospinal fluid from the surrounding does not readily occur [7]. In our two patients, AEDH without fracture was formed in the frontal region due to contusion in the occipital region, and the source of bleeding was the middle meningeal artery. It was assumed that negative pressure was produced in the frontal region by contusion in the occipital region and damaged the dura mater leading to breakage of the middle meningeal artery even though no fracture occurred.

Contrecoup AEDH was not detected on the first head CT and it was initially discovered on the second imaging in 8 (38%) of the 21 cases, a high rate. Moreover, delayed hematoma formation occurred after 10 h and 2.5 days in two of the three fatal cases, respectively. Based on these findings, careful course observation and time-course evaluation by imaging should be performed in consideration of delayed AEDH formation in regions other than the impact point even though no fracture is observed.

## Conclusion

Two rare cases of contrecoup frontal AEDH without fracture near the hematoma were reported. According to previous reports, the incidence of this type of AEDH is high in people in their 50s. Regarding the developmental mechanism, it was assumed that the dura mater was detached from the inner surface of the skull due to negative pressure induced by the cavitation theory, and blood vessels in the dura mater were injured and caused hemorrhage. Since AEDH may develop on the contralateral side of the impact point even though no fracture is present, it may be important to perform imaging examination over time to avoid overlooking the formation and enlargement of hematoma.

## Abbreviations

AEDH: Acute epidural hematoma
GCS: Glasgow Coma Scale
